# Letter from Dr. Fellger

**Published:** 1882-05

**Authors:** Ad. Fellger

**Affiliations:** Philad’a


### LETTER FROM DR. FELLGER.

*Editor* HOMŒOPATHIC PHYSICIAN:

DEAR DOCTOR: As you cannot indorse all that Prof. Butlerow advances in his letter [See p. 184—ED. H. P.], I assure you decidedly that I can do so, every word of it; and as you see that our opinions so widely differ, I would advise you to leave my name off as contributor to THE HOMŒOPATHIC PHYSICIAN, as I fear under such circumstances, a conflict between us two, sooner or later, could not be avoided. Truly yours,

*Philad'a, April 17th.* AD. FELLGER.

